# Advance in pediatric spinal cord injury

**DOI:** 10.1002/pdi3.55

**Published:** 2024-03-22

**Authors:** Jian Zheng, Guoxin Nan

**Affiliations:** ^1^ Dongguan Children's Hospital Affiliated to Guangdong Medical University Dongguan China; ^2^ Pediatric Research Institute Children's Hospital of Chongqing Medical University Chongqing China; ^3^ Dongguan Eighth People's Hospital Dongguan China; ^4^ Dongguan Institute of Pediatrics Dongguan China

**Keywords:** pediatric spinal cord injury, SCIWORA, spinal cord injury, spine

## Abstract

The incidence rate of spinal cord injury in children is lower than that in adults, accounting for about 5% of all spinal cord injuries. Motor vehicle accidents are the main cause of spinal cord injuries in children. As the spine of children is still in the process of growth and development, the anatomical structure and biomechanics have unique characteristics, and its etiology, injury site, and clinical manifestations are different from those of adults. Misdiagnosis and delayed diagnosis can lead to severe spinal deformity and neurological complications. Children and adolescents with spinal cord injuries may suffer from lifelong disability, which will do great harm to children, families, and society. Early magnetic resonance imaging examination can effectively avoid underdiagnosis of spinal cord injury without radiographic abnormality and select appropriate treatment. In addition, it is also important to establish a family‐centered rehabilitation model to help the affected children reintegrate into society and achieve the goal of returning to normal life. This article reviews the etiology, epidemiology, clinical characteristics, complications, and treatment of spinal cord injury in children and adolescents.

## 1 INTRODUCTION

Spinal cord injury (SCI) is a serious traumatic disease of the central nervous system caused by external direct or indirect factors, and the corresponding changes of various motor, sensory and sphincter dysfunction, dystonia, and pathological reflexes occur in the corresponding segments of the injury.^1^ SCI is a devastating injury that can have serious consequences for both adults and children, especially for children and adolescents who have little chance of experiencing childhood.

There are two types of spinal cord injuries: primary and secondary.^1^ The injury caused by external forces acting directly or indirectly on the spinal cord is referred to as primary spinal cord injury. Physiological changes that occur hours or days after the initial injury cause secondary spinal cord injury. The mechanisms of secondary injury include ischemia, increased intracellular calcium, extracellular glutamate, free radicals, inflammation, and apoptosis. After the primary injury, a series of progressive secondary injury cascade reactions characterized by ischemia, apoptosis‐promoting signals and peripheral inflammatory cell infiltration were initiated. First, due to the destruction of the blood–spinal cord barrier, inflammatory cells, such as macrophages, T cells, microglia, and neutrophils, infiltrate the injured site and release inflammatory factors.^2^ In addition, an imbalance of ion homeostasis will occur after spinal cord injury, leading to high intracellular calcium, activating calcium‐dependent proteases, mitochondrial dysfunction and ultimate apoptosis.^3^ Moreover, after spinal cord injury, phagocytic inflammatory cells release reactive oxygen species, leading to DNA oxidative damage, protein oxidation, and lipid peroxidation, further inducing nerve necrosis and apoptosis.^4^ Finally, due to the release of damaged cells, excitatory amino acids (glutamic acid and aspartic acid) increase.^5^, ^6^ The excessive activation of excitatory amino acid receptors produces excitotoxicity and further expands the loss of neurons and glial cells through necrosis and apoptotic cell death.^7^ This chain of events enlarges the area of nerve tissue injury and worsens the neurological function defect and prognosis.^8^, ^9^

Furthermore, there are numerous variables that impede nerve regeneration following a spinal cord injury. Due to the death and loss of a large number of cells in the focal center during the middle and chronic stages of spinal cord injury, cystic microvacuoles form and multiple microvacuoles merge into cystic cavities, which are then wrapped in fibrous connective tissue, becoming an important impediment to cell migration and axon regeneration.^10^ Hypertrophic astrocytes form a physical barrier for glial scars to separate damaged tissue from healthy tissue following a spinal cord injury. By secreting significant amounts of growth‐inhibiting chondroitin sulfate proteoglycans (CSPGs), astrocytes also build a chemical barrier to impede spinal cord regeneration.^11^

SCI in children is still a relatively rare condition compared to its prevalence in adults. However, up to 5% of SCI still occur in children.^12^ The etiology, injury site, and treatment of SCI in children differ from those in adults due to the unique biomechanics and anatomy of the spinal column in children at their stage of growth and development.^13^

This article reviews the etiology, epidemiology, clinical features, complications and management of spinal cord injury in children and adolescents.

## 2 ETIOLOGY AND EPIDEMIOLOGY

The main causes of spinal cord injury include falls, sports accidents, and motor vehicle accidents. The mechanism of injury varies with the age of the patient. Fall injury is a common cause of spinal cord injury in adults, but it is uncommon in children, with the majority occurring in children under the age of 8.^14^ In China, sports accidents are the leading cause of spinal cord injuries in children, predisposing them to thoracic spine injuries.^15^ Around the globe, motor vehicle accidents are the leading cause of spinal cord injuries in children, usually affecting younger children, and are the most common cause of cervical spine injuries in children, accounting for 48%–61% of injuries in all age groups.^13^, ^16^ Compared with adult patients, cervical spine injury accounts for 30%–40% of adult spinal trauma, but in pediatric patients, cervical spine injury accounts for 80% of spinal cord injury.^17^

The anatomical structure of the developing child's spine makes the cervical vertebra more likely to be injured. Before the age of 8, the head of the child is relatively heavy compared with the total body weight, while the neck muscles of the child are still weak. The cervical vertebra is wedge‐shaped in front, and the spinous process, transverse process and uncinate process are underdeveloped.^18^ When a car accident occurs, the head moves violently, which can result in cervical injury. For younger children, when the head is moving, the movement fulcrum is in the upper cervical vertebra and the maximum movement is in the C2/3 (the second and third cervical vertebrae) segment, which makes the younger children prone to damaging the upper cervical vertebra. With age, the weight and size of the head become smaller relative to other parts of the body, and the fulcrum moves down to the C5/6 (the fifth and sixth cervical vertebrae) segment, which continues until adulthood. Therefore, older children and adults usually suffer lower cervical spine injuries.^19^, ^20^

In addition, there are some unique causes of SCI in children, such as birth injuries and lap belt injuries.^21^ One in every 60,000 newborns suffers a spinal cord injury.^22^ Abnormal position of the newborn during delivery, prolonged delivery time, forceps‐assisted delivery, and shoulder dystocia can all lead to cervical spine injury in the newborn, leading to relatively severe neonatal respiratory distress and hypotonia with soft paraplegia or quadriplegia.^23^ Lap belt injuries are often caused by motor vehicle accidents and often affect children with small bodies because the seat belt is higher than the pelvic rim and acts as a front fulcrum, resulting in flexion and stretch forces in the middle of the lumbar spine.^24^ The main causes of lap belt injuries are abdominal wall bruising, abdominal viscera injury, and lumbar spine injury. The abdominal wall bruising is caused by a seat belt injury, the most common visceral injury is a perforation or tear in the small or large intestine, and lumbar spine injury are the most common between L2 (the second lumbar vertebra) and L4 (the fourth lumbar vertebra). In addition, 23%–30% of children with lap belt injuries had spinal cord injury without radiographic abnormalities. Although the force of the belt injury was concentrated in the mid‐lumbar spine, nerve damage varied from the mid‐chest to the conus or cauda equina.^24^

Overall, the cervical spine (87.2%) was the most common site of SCI in children, followed by the thoracic spine (9.6%) and the lumbar spine (1.5%).^25^

### 2.1 Clinical characteristics and complications

Some children with spinal cord injuries are unable to see spinal fracture or instability on plain film or CT. This is called spinal cord injury without radiographic abnormalities (SCIWORA), first described by Pang et al. in 1982.^26^ SCIWORA is much more common in children and adolescents, accounting for 74.1% of SCI incidence in children and adolescents.^15^ The unique anatomical and physiological features of prepubertal children are responsible for SCIWORA, and the incidence of SCIWORA decreases with age and becomes less common in adulthood. About 64% of children aged 5 years or younger at the time of injury have SCIWORA, while only 32% of those aged 6–12 years have it. SCIWORA is less common in thoracic spine injury and more common in cervical spine injury.^27^ About 40% of children with SCIWORA suffer from paresthesia, lower limb pain, and general paralysis within 30 min to 4 days after injury.^28^ Early diagnosis of SCIWORA is difficult because of the young age of children, their inability to express these symptoms of nerve injury through language, and the difficulty of cooperating with relevant physical examination.^29^ However, with the increased use of magnetic resonance imaging (MRI), early stages of SCIWORA can be easily identified. T1‐weighted imaging can show the shape and anatomy of the spinal cord, while T2‐weighted imaging (low T2 signal intensity) can identify the bleeding site. Diffusion‐weighted imaging (DWI) can detect structural damage and neurological prognosis by evaluating the integrity of the white tract of the spinal cord.^30^, ^31^ As a result, in children with SCI, early MRI scanning can assist in better evaluating the situation and avoid missing the best treatment time window, which is advantageous to spinal cord injury prognosis.

Compared with adults, children with SCI are more likely to develop paraplegia or complete lesions.^32^ Although paraplegia and complete injury occur in approximately two‐thirds of children with SCI, the incidence of these injuries decreases with age, with paraplegia and complete injury occurring in only 45% of adult patients.^33^ However, some studies have suggested that, compared with adults, children's neurological function will recover better, and the recovery of an incomplete injury is better than that of a complete one.^14^

Furthermore, children are predisposed to orthopedic complications such as spinal deformities (or scoliosis) and hip deformities.^34^ When children's skeletal development is not complete, spinal deformity is easy to occur when they are injured by the spinal cord at this time, most of which will eventually develop into scoliosis. The degree of scoliosis in most patients is 40°, and the degree of scoliosis in patients with a complete injury will exceed 40°.^29^ Sixty‐seven percent of these children with scoliosis required surgical treatment. In contrast, scoliosis is very rare in adults, and only 5% of adult patients require surgical correction. Children with paraplegia and complete injuries are significantly more likely to have hip deformities.^29^ There is a certain relationship between the incidence of hip deformity and the age of children. According to the study results, hip instability can occur in about 66% of children under the age of 4 who have a spinal cord injury. While only 6% of spinal cord injury patients between the ages of 13 and 21 reported hip instability, the opposite was true for other patients.^35^ The surgical indications for hip instability are not clear. For children under 10 years with hip instability, soft tissue stretching, control of spasticity, and other measures are recommended to prevent it. Severe spasticity can be controlled with drugs.^34^

In addition to orthopedic complications, autonomic dysreflexia and hypercalcemia are also common in children with spinal cord injury. Autonomic dysreflexia is the most dangerous complication, which occurs in SCI patients with T6 or above.^36^ Stimulation lower than the injured part causes sympathetic nerve overreaction. The sympathetic nerve is not inhibited by the brain center, resulting in high blood pressure, headache, arrhythmia, red skin, blurred vision and other symptoms in adults and children, and lethargy or irritability in younger infants.^37^ The difference is that the baseline level of children's blood pressure is low, and children's noncooperation makes it difficult to detect blood pressure, which may miss the best treatment time. Therefore, it is very important to measure blood pressure regularly.^36^ Hypercalcemia often occurs within 3 months after injury, with an incidence of 10%–23%, which is caused by increased bone resorption.^38^ Children and adolescents are in the growth stage, with increased bone turnover and more bone mass with active metabolism, which makes children and adolescents with spinal cord injury prone to hypercalcemia.^39^ Patients with hypercalcemia usually show abdominal pain, nausea, vomiting, fatigue, lethargy, polyuria, polydipsia, and dehydration. Furosemide can be used for treatment.^40^

## 3 TREATMENT

Following a spinal cord injury, the spinal cord's ability to repair itself is limited, and there is currently no effective treatment for the injured spinal cord. The principles of spinal cord injury treatment and necessity in children are similar to those in adults.^41^ Children continue to grow and develop as well as their unique manifestations and complications, and treatment must be based on development and tailored to individual special needs. The main goal of treatment is to decompress the neural elements and stabilize the spine in order to prevent further spinal cord injury.^16^ The current treatment methods include conservative treatment and surgical treatment. Conservative treatment mainly includes drug therapy. However, neither conservative treatment nor surgical treatment can completely restore the injured nerve function. Cell transplantation and biomaterials are new therapeutic strategies under research and development.

### 3.1 Medication

Drug treatment is a conservative treatment, and the most commonly used drug is methylprednisolone（MP）. However, whether MP can be used to treat patients with SCI is still controversial.^42^

In the treatment of adult patients, MP has a beneficial effect on SCI if given within the 8‐h therapeutic window after injury.^43^ “Several studies have demonstrated a beneficial effect of MP on neurologic outcomes, but its beneficial effect is uncertain, and these reports have not clearly demonstrated neurologic improvements of a functional or behavioral nature.” After treatment with MP, some patients show improvement in sensory function but no improvement in motor function,^27^ and some patients show improvement in motor function but no improvement in sensory function.^44^, ^45^ However, in patients beyond the 8‐h treatment window, MP use had a detrimental effect on neurological recovery.^46^ In addition, the incidences of wound infection, gastrointestinal complications, and pulmonary embolism caused by MP use were also higher.^42^ Therefore, MP should not be routinely used in adults with SCI.

The use of MP in children is also controversial, especially the dosage.^32^ The efficacy of MP was first reported in 1990 by Bracken et al., in which MP was administered orally at 30 mg/kg followed by an hourly infusion of 5.4 mg/kg for 23 h.^47^ Although this approach has been used in children and adolescents, the youngest patient in this study was 13 years of age, and it is unclear whether this approach is applicable to younger children. Similarly, the use of MP also increases the risk of complications in children and adolescents, and it is a proven fact that the use of high‐dose steroid hormones increases the risk of complications. In a study by Cage et al. in 2015, 23 children in the treatment group (mean age 6.6 years) were treated with high‐dose steroid hormones, and the results showed that the incidence of hyperglycemia and respiratory tract infection in the treatment group was significantly higher than that in the control group (without steroid hormone treatment).^48^ Despite differences in the treatment of children and adolescents with SCI and high‐dose MP, it remains the drug of choice in the absence of an effective treatment at this stage.

In addition to MP, minocycline and cyclosporine A have been used in patients with SCI, both of which inhibit secondary damage.^49^ However, the use of these drugs remains controversial as none of them have shown beneficial neurological recovery results and have even led to some adverse effects.^50^, ^51^

### 3.2 Surgery

The secondary reaction of a SCI aggravates the injury site. Early surgical decompression can improve the microenvironment, reduce the pressure in the microvascular circulation, and reduce hypoxia and ischemia.^52^ Neurological recovery is aided by decompression surgery performed within 24 h of injury. Surgical removal of bone fragments and soft tissue fragments at the injury site can reduce compression symptoms related to secondary edema, and surgical methods of fusion and internal fixation can restore the anatomical alignment of the spine and reduce the risk of secondary injury, which is beneficial to nursing and rehabilitation sports.^53^, ^54^ In cases where the neurological injury still has good healing potential, decompression alone can be performed, and the anatomical reduction of the spine can be monitored for a long time. In cases of neurological instability and poor chances of long‐term healing, decompression and fusion are often performed simultaneously.^55^

Whether children with SCI need surgical treatment depends not only on the type of fracture but also on the degree of nerve damage at the injury site.^55^ Stable fractures can be treated conservatively, while unstable fractures require surgical stabilization. Cervical spine injury in children generally do not require surgical intervention and external fixation orthopedic devices can be selected for treatment. If the type of cervical spine fracture is an atlanto‐occipital joint dislocation, burst fracture, compression fracture with deformity, or fracture or dislocation with compression symptoms, surgical intervention may be required.^56^ Anterior or posterior fusion can be used for surgery, and older children can use plates and screws as auxiliary means of fusion. After surgery, a cast or halo immobilization is needed for an average of 150 days.^57^ Children with thoracic and lumbar injury with stable fractures can choose an orthosis and brace for fixation under conservative treatment, and the fixation time is generally 3 months.^58^ Burst fractures can also be treated with a stent if there is no nerve damage. Posterior instrumentation and fusion are often selected for surgical treatment, and reduction and stabilization can be performed in one level above and below the fracture or in two levels above and below the injury according to the degree of injury and spinal instability.^59^ To prevent the risk of spinal deformity, external fixation with a brace is recommended after surgery, and regular physical examinations should be performed after surgery into adulthood.

### 3.3 Cell transplantation and biomaterial

Cell‐based regenerative therapies show promise for the treatment of SCI (Table 1). Studies have reported that the cells that can be used for cell transplantation include olfactory ensheathing cells, neural stem cells, mesenchymal stem cells, and Schwann cells.^71^ These cells for transplantation can secrete neurotrophic factors, such as mesenchymal stem cells and Schwann cells, which may potentially enhance cell activity, improve the microenvironment, promote angiogenesis, and play a neuroprotective role.^72^, ^73^

**TABLE 1:** Cell types and functions.

Type	Function
Olfactory ensheathing cells	Neuroprotection^60^
	Axon regeneration^61^
Schwann cells	Neuroprotection^62^
	Axon regeneration^63^
	Myelinogenesis^64^
Mesenchymal stem cells	Neuroprotection^65^
	Axon regeneration^66^
	Immunomodulation^67^
Neural stem cell	Neuroprotection^68^
	Axon regeneration^69^, ^73^
	Myelinogenesis^69^
	Immunomodulation^70^

Transplanted cells can also secrete cytokines that regulate inflammation, reduce harmful inflammation, stimulate beneficial inflammation, and also produce beneficial effects on repair.^74^ In addition, remyelination is a process in which transplanted cells generate myelin around demyelinated axons, and demyelinated axons can be remyelinated through cell transplantation to achieve functional improvement.^75^ Finally, the most important thing about whether cell transplantation is effective is whether the transplanted cells are able to promote axonal regeneration. The axons of the spinal cord at the injury site are interrupted, and a multicellular structure is formed through cell transplantation. When the transplanted cells can differentiate into neurons and form synapses with the host neurons, the descending axons will form connections with the newly differentiated transplanted neurons, promoting the regeneration of the interrupted axons.^71^, ^76^

However, single‐cell transplantation has certain shortcomings; the transplanted cells cannot maintain the stability of tissue structure at the site of injury, and the cells move to different places. At the same time, due to the existence of a large number of inflammatory cells and inflammatory factors at the injured site, the survival and proliferation of cells are not conducive and the expected therapeutic effect may not be fully achieved.^77^ The use of biomaterials solves these problems. Living cells are wrapped in biological materials and collectively transplanted to the injured site (Table 2). The biological materials can separate the cells from the surrounding pathological microenvironment and provide a microenvironment conducive to cell survival, proliferation, and differentiation so that the cells can have a better repair effect.^81^ In addition, biomaterials fill the cavities at the site of injury, connect the spinal stump, and provide physical and directional support for axon regeneration.^82^ Multiple studies have shown that combined transplantation is beneficial for SCI repair. In Olson et al.,^83^ neural stem cells and Schwann cells were loaded on a poly‐co‐glycolic acid polymer scaffold (PLGA) to treat rats with spinal cord transection injuries. When cultured in vitro and in vivo, both neural stem cells and Schwann cells can survive, proliferate, and reproduce in PLGA and have a good therapeutic effect on the transected spinal cord. The research team of Zeng X used a three‐dimensional gelatin scaffold with bone marrow mesenchymal stem cells and found that they played an important role in reducing inflammation, promoting angiogenesis, and reducing cavity formation in rats with spinal cord transection injuries.^84^

**TABLE 2:** Joint transplantation of cells and biomaterials.

Cell	Biomaterial	Model	Result
Neural stem cells	Hydrogel	Complete transection of rat spinal cord	More axon growth was observed in the scaffold channel, and the survival rate of neural stem cells was improved.^78^
Neural stem cells	Collagen	Complete transection of rat spinal cord	Reduced cyst formation at the site of injury.^79^
Schwann cells	Gelatin methacryloyl hydrogel	Lateral hemisection at T10	It improved functional recovery and significantly inhibited apoptosis after SCI.^80^
Mesenchymal stem cells	Hydrogel	Complete transection of rat spinal cord	It promotes the differentiation and extension of neurons in vitro.^67^

Despite the good results of cell transplantation in animal experiments, there are still some problems with this therapeutic approach. Most of the current research has been done on animal models, and there is little clinical data on its use in humans. Children and adolescents are a special group, and the long‐term safety and efficacy of cell transplantation are still unclear. As children and adolescents grow, it is unknown whether early transplantation treatments still have a role to play and whether they may have adverse effects such as immune rejection and tumor formation. In addition, although the current use of biomaterials has shown therapeutic potential in animal models and different cells in combination with different biomaterials have different therapeutic effects, whether they have the ability to improve neurological prognosis in humans remains elusive.^85^ Most of the experimental models used in the existing experimental results are rodents, and few clinical trials in adults and children have been reported, but the results already achieved give us hope that the combined transplantation of biomaterials and cells will bring us surprises in the future as more clinical trials are conducted.

### 3.4 Functional electrical stimulation

As early as the 1960s, electrical stimulation of the peroneal nerve was used to stimulate muscle function in the lower limbs and improve foot drop symptoms in stroke patients with hemiplegia, whcih is one of the earliest applications of electrical stimulation.^86^ Functional electrical stimulation (FES) is a type of neuromuscular stimulation in which electrical stimulation is applied to muscles or nerves to achieve functional and purposeful movements, such as holding a pen to write or lifting a glass of water.^87^ FES and other types of neuromuscular electrical stimulation have been shown to improve circulation, range of motion, muscle strength, and muscle spasticity.^88^ FES delivers stimulation through electrodes that can be attached to the surface of the skin or implanted inside the body. By adjusting the position of the electrodes, stimulation can be targeted to specific muscle groups, allowing for targeted exercise training or rehabilitation.^89^ FES is often used in neuroprostheses that can help patients perform various movements, including standing, walking, grasping, etc. FES can be used to activate the muscles around the ankle, which in combination with a standing frame to support the trunk, can restore the ability to stand. Parastep is an FDA‐approved device that stimulates the peroneal nerve and lower limb muscles through implanted neuroprostheses, allowing SCI patients to walk short distances.^90^

Moineau et al.^91^ collaborated with Myant Inc. (Toronto, Canada) to develop functional electrical stimulation garments that are easy to put on and take off, can be customized, are cost‐effective, versatile, durable, and allow patient independence. The garment design combines fabric with silver wires or conductive layers and electrodes embedded in the garment to create a highly conformable structure that delivers electrical stimulation. The electrical stimulation can be delivered by controlling the strength and frequency of the current to produce specific movements on the wearer's body. Although the garment has only been tested on healthy participants, its potential feasibility gives hope to spinal cord injury patients who wish to have autonomous mobility.

FES is already an important tool in the field of neurorehabilitation therapy, and the applications of electrical stimulation will continue to expand as technology and interventions evolve. Neuroplasticity induced by electrical stimulation offers the promise of therapeutic interventions that can improve the quality of life of patients with spinal cord injury. The prevalence of electrical stimulation is likely to increase in the future, and neuroprosthetic devices will play an important role in rehabilitation.

## 4 NEUROTROPHIC SUPPORT

The challenging microenvironment following spinal cord injury severely hampers the recovery of neurological function and leads to increased complexity of treatment. The enhancement of neurotrophic support functions can create a favorable microenvironment, thereby facilitating spinal cord recovery. Neurotrophic factors (NFs) enhance the survival of neurons and synaptic plasticity.^92^ Studies indicate that there is a decreased expression of NFs after sustaining spinal cord injuries. A possible solution is to increase the levels of NFs through multiple injections, continuous infusion, or corelease in combination with biomaterials. This method may create a more propitious environment, which can promote neural regeneration and alleviate functional deficits resulting from SCI.^93^ Delivery of neuronal growth factor (NGF) to the injured spinal cord through a delivery system based on nanocapsules suppressed the inflammatory response after spinal cord damage and stimulated neuronal regeneration in a mouse model of SCI.^94^ Subcutaneous injection of fibroblast growth factor 2 (FGF2) inhibited TNF‐a expression, attenuated monocyte/macrophage infiltration, and glial proliferation in the lesions of mice suffering from spinal cord injury. Subsequently, functional recovery of the paralyzed hind limbs in the mice was improved.^95^ Neurotrophic factor‐3 (NT‐3)/silk cardiac myosin complexes supported by gelatin sponge scaffolds were transplanted into a canine spinal cord half‐cut model. The results inhibited local inflammation, promoted nerve fiber regeneration, and improved nerve conduction in dogs.^96^ The enhanced neurotrophic support yielded better results in animal models and paves the way for a new avenue of treatment for central nervous system injuries.

## 5 CONCLUSION

The origin, clinical characteristics, and management of spinal cord damage differ between children and adults. Although the incidence of SCI in children is lower and neurological function recovery ability may be better than in adults, the treatment of children still faces hurdles and requires specific planning due to children's unique anatomical structure and continuous growth and development. Include therapy objectives that evolve as the child matures. When compared to adults, the incidence of spinal cord damage in children is rather low. However, it is difficult to diagnose SCI in youngsters, and special attention should be paid to the early stages of injury. To minimize misdiagnosis of SCIWORA, MRI detection should be performed as soon as possible. Although the anatomic compliance of a kid's spine provides protection, high intensity damage pressures that surpass the spine's tolerance generate lasting and traumatic injury with profound psychological and physical consequences for the child. Current treatments do not fully restore nerve function, and breakthroughs in cell transplants and biomaterials are possible treatments that, while few human trials have been undertaken, offer optimism that SCI can be treated. The ultimate goal of healing children with spinal cord injuries is to enable them to become adults by constructing dynamic, appropriate rehabilitation programs centered on the family, enabling children to constantly adapt to changing physiological conditions and gradually become independent, helping them to reintegrate into society and lead a normal life. Of course, prevention is also very important. Appropriate protective measures can well prevent this disease, such as the installation of a car seat, which can effectively reduce the incidence of motor vehicle accidents caused by spinal cord injury. Children in sports need to do a good job of protection, best accompanied by parents. SCI is still a challenging disease, and with increasing data from clinical trials, it may be possible to cure it someday.

## AUTHOR CONTRIBUTION

Guoxin Nan conceived and designed the study. Jian Zheng drafted the manuscript and made drawings. All the authors read and approved the final manuscript

## CONFLICT OF INTEREST STATEMENT

The authors declare that there are no conflict of interest.

## ETHICS STATEMENT

None

## Data Availability

Data sharing is not applicable to this article as no datasets were generated or analyzed during the current study.
